# Supplementary material for: Blackjack mutations improve the on-target activities of increased fidelity variants of SpCas9 with 5′G-extended sgRNAs
Source: Nat Commun. 2020 Mar 6;11:1223. doi: 10.1038/s41467-020-15021-5 (PMC7060260; doi:10.1038/s41467-020-15021-5)
Supplement: Supplementary file 9 — Description of Additional Supplementary Files [file 41467_2020_15021_MOESM9_ESM.pdf]

Title: Supplementary Data 1

Description: This file, which has multiple tabs, contains sgRNA, primer and sequence data.

Title: Supplementary Data 2

Description: This file contains the summary of targeted deep-sequencing data.

Title: Supplementary Data 3

Description: This file, which has multiple tabs, contains the summary of GUIDE-seq data.

Title: Supplementary Data 4

Description: This file, which has multiple tabs, contains data of the Figures.

Title: Supplementary Data 5

Description: This file, which has multiple tabs, contains data of the Supplementary Figures.

Title: Supplementary Data 6

Description: This file, which has multiple tabs, contains summaries of the data distributions and statistical details related to each of the manuscript Figures.
