# Supplementary material for: Blackjack mutations improve the on-target activities of increased fidelity variants of SpCas9 with 5′G-extended sgRNAs
Source: Nat Commun. 2020 Mar 6;11:1223. doi: 10.1038/s41467-020-15021-5 (PMC7060260; doi:10.1038/s41467-020-15021-5)
Supplement: Supplementary file 10 — Reporting Summary [file 41467_2020_15021_MOESM10_ESM.pdf]

nature research

Corresponding author(s): Ervin Welker

Last updated by author(s): Jun 25, 2019

Reporting Summary

Nature Research wishes to improve the reproducibility of the work that we publish. This form provides structure for consistency and transparency in reporting. For further information on Nature Research policies, see Authors & Referees and the Editorial Policy Checklist .

Statistics

For all statistical analyses, confirm that the following items are present in the figure legend, table legend, main text, or Methods section.

n/a	Confirmed	
☐	☒	The exact sample size (*n*) for each experimental group/condition, given as a discrete number and unit of measurement
☐	☒	A statement on whether measurements were taken from distinct samples or whether the same sample was measured repeatedly
☐	☒	The statistical test(s) used AND whether they are one- or two-sided<br>*Only common tests should be described solely by name; describe more complex techniques in the Methods section.*
☒	☐	A description of all covariates tested
☐	☒	A description of any assumptions or corrections, such as tests of normality and adjustment for multiple comparisons
☐	☒	A full description of the statistical parameters including central tendency (e.g. means) or other basic estimates (e.g. regression coefficient) AND variation (e.g. standard deviation) or associated estimates of uncertainty (e.g. confidence intervals)
☐	☒	For null hypothesis testing, the test statistic (e.g. *F*, *t*, *r*) with confidence intervals, effect sizes, degrees of freedom and *P* value noted<br>*Give P values as exact values whenever suitable.*
☒	☐	For Bayesian analysis, information on the choice of priors and Markov chain Monte Carlo settings
☒	☐	For hierarchical and complex designs, identification of the appropriate level for tests and full reporting of outcomes
☒	☐	Estimates of effect sizes (e.g. Cohen's *d*, Pearson's *r*), indicating how they were calculated

Our web collection on statistics for biologists contains articles on many of the points above.

Software and code

Policy information about availability of computer code

Data collection	Images from western-blot experiments were taken using Chemidoc MP (Bio-Rad) with Image Lab 4.1 Software (Bio-Rad). Flow cytometry data were collected on Attune NxT Acoustic Focusing Cytometer (Applied Biosystems) using Attune NxT Software v.2.7.0.
Data analysis	GUIDE-seq data were analysed using the pipeline kindly provided by J. Kieth Joung, Martin J Aryee and Shengdar Q. Tsai (open-source guideseq software version 1.1; https://github.com/aryeelab/guideseq). Targeted deep-sequencing data were analysed using a the following softwares: BBMap 38.08, samtools 1.8, BioPython 1.71, PySam 0.13. Statistical analyses were performed using IBM SPSS (ver. 20). Some of the indel efficiencies were analyzed by TIDE webtool (https://tide.nki.nl/). Flow cytometry data were futher analyzed using Attune NxT Software v.2.7.0.

For manuscripts utilizing custom algorithms or software that are central to the research but not yet described in published literature, software must be made available to editors/reviewers. We strongly encourage code deposition in a community repository (e.g. GitHub). See the Nature Research guidelines for submitting code & software for further information.

Data

Policy information about availability of data

All manuscripts must include a data availability statement. This statement should provide the following information, where applicable:

- Accession codes, unique identifiers, or web links for publicly available datasets
- A list of figures that have associated raw data
- A description of any restrictions on data availability

Important plasmids used in this study are available at Addgene (details in Plasmid Construction section). Sequences of the constructs are listed in Supplementary information. All associated raw data is available in Supplementary Data 4 and 5 for Fig. 1-7 and Supplementary Fig. 1-8, respectively; Supplementary Data 3 for Fig. 3b, 5f and Supplementary Fig 5, 7. The data that support the findings of this study are available from the corresponding authors upon request as well. The deep sequencing data (targeted deep-sequencing and GUIDE-seq) from this study have been submitted to the NCBI Sequence Read Archive (SRA; http://www.ncbi.nlm.nih.gov/sra/) under accession number: PRJNA593843.

Field-specific reporting

Please select the one below that is the best fit for your research. If you are not sure, read the appropriate sections before making your selection.

- [x] Life sciences
- [ ] Behavioural & social sciences
- [ ] Ecological, evolutionary & environmental sciences

For a reference copy of the document with all sections, see nature.com/documents/nr-reporting-summary-flat.pdf

Life sciences study design

All studies must disclose on these points even when the disclosure is negative.

Sample size	No statistical methods were used to predetermine or justify sample sizes, but each condition was performed in triplicate which is a generally accepted sample size for such studies.
Data exclusions	No data were excluded.
Replication	All attempts at replication were successful.
Randomization	The experiments were not randomized.
Blinding	The investigators were not blinded to allocation during experiments and outcome assessment.

Reporting for specific materials, systems and methods

We require information from authors about some types of materials, experimental systems and methods used in many studies. Here, indicate whether each material, system or method listed is relevant to your study. If you are not sure if a list item applies to your research, read the appropriate section before selecting a response.

Materials & experimental systems

n/a	Involved in the study	
☐	☒	Antibodies
☐	☒	Eukaryotic cell lines
☒	☐	Palaeontology
☒	☐	Animals and other organisms
☒	☐	Human research participants
☒	☐	Clinical data

Methods

n/a	Involved in the study	
☒	☐	ChIP-seq
☒	☐	Flow cytometry
☒	☐	MRI-based neuroimaging

Antibodies

Antibodies used	anti-FLAG (F1804, Sigma); anti-β-actin (A1978, Sigma); HRP-conjugated secondary anti-mouse antibody (715-035-151, Jackson ImmunoResearch)
Validation	*Describe the validation of each primary antibody for the species and application, noting any validation statements on the manufacturer's website, relevant citations, antibody profiles in online databases, or data provided in the manuscript.*

Eukaryotic cell lines

Policy information about cell lines

Cell line source(s)	N2a cell line and 293T were obtained from ATCC, 293-H from Gibco, N2a.dd-EGFP, N2a.EGFP and HEK-293.EGFP were cloned by us.
Authentication	Cell lines were not authenticated as they were obtained directly from a certified repository or clone from those cell lines.
Mycoplasma contamination	Cells were tested monthly for mycoplasma contamination with negative test results.
Commonly misidentified lines (See ICLAC register)	No commonly misidentified cell lines were used.
